# Relationship of the orange tissue morphotype with shell and pearl colouration in the mollusc *Pinctada margaritifera*

**DOI:** 10.1038/s41598-019-41581-8

**Published:** 2019-03-26

**Authors:** Chin-Long Ky, Carole Blay, Floriane Broustal, Manaarii Sham Koua, Serge Planes

**Affiliations:** 1 Ifremer, UMR 241 EIO, Labex Corail, Centre du Pacifique, BP 49, 98719 Taravao, Tahiti, Polynésie Française, France; 2 PSL Research University, EPHE-UPVD-CNRS, USR 3278 CRIOBE, Labex Corail, Université de Perpignan, 52 Avenue Paul Alduy, 66860 Perpignan Cedex, France

## Abstract

Molluscs display a vast range of shell colours both between and within species. However, only a few species show colour variation in their soft tissues. In French Polynesia, the pearl oyster *Pinctada margaritifera* has three tissue morphotypes: the black wild-type and two rare mutations: white albino and orange mantle. Phenotypic transmission is known to occur from these phenotypes when they are used as graft donors for pearl production, leading to multicoloured and white pearls from black and albino mantle grafts, respectively. The present study furthers this knowledge by examining the phenotypic association between the orange mantle tissue morphotype and hard tissues: shells and cultured pearls. Based on a large experimental graft, shell colour quantification and pearl qualification showed that the orange morphotype is associated with light-coloured shells and pearls. Expression analysis of some candidate genes previously identified in the white mantle mutant, tested here on both graft and pearl sac tissues from orange mantle donors, confirmed the involvement of genes associated with shell matrix protein (*shem4*) and the melanin biosynthesis pathway (*zinc*). This study provides fundamental information on the mechanism behind mantle tissue colour in *P. margaritifera* and its association with biomineralisation and pigmentation processes that will be potentially valuable in future selection programs.

## Introduction

The pearl oyster *Pinctada margaritifera* (Bivalvia, Mollusca) is an economically, ecologically and biologically important species, widely distributed in the Indo-Pacific region^1,2^. It is also an interesting animal transplant model for the study of phenotypic transmission, particularly for colour traits^3^. This species is used to produce cultured pearls, which are formed by the biomineralization process of a piece of grafted mantle tissue from a donor oyster inserted, together with a nucleus, into the gonad of a recipient oyster^4,5^. Following grafting, a pearl sac forms composed of chimeric tissue containing the two genomes. Pearl quality traits are determined by complex interactions between these recipient and donor genomes and the environment^6,7^. Recent studies have examined the contribution of donor and recipient oysters to the transmission and expression of phenotypic traits, and performed overall phenome screening^3,8^. In comparison with other *Pinctada* species, the colour range of cultured pearls from *P. margaritifera* overlaps those produced by both *P. maxima* and *P. fucata*. The silver-lip or gold-lip pearl oyster, *P. maxima*, farmed mostly in Indonesia, Australia, the Philippines and Myanmar, produces light-coloured pearls described as “white”, but which include yellow, cream, golden and light grey hues^9^. The “Akoya pearls” produced in Japan and China by *P. fucata* are also light in colour and may be pink, white, cream, yellow, or even silver^10^. By contrast, the black-lip pearl oyster, *P. margaritifera*, is famed for its production of dark pearls, which show different combinations of main bodycolor and secondary colour. *P. margaritifera* pearls may vary from the purest white to the deepest black, passing through every shade of silver, peacock, green, aubergine, purple, golden brown and even rainbow^11,12^. Pearl production with this species is mainly concentrated in French Polynesia. Other countries of the Pacific region, such as the Cook Islands, Fiji islands and Micronesia, have also developed pearl farming industries based on *P. margaritifera* and the species is also cultured in the Indian Ocean, specifically in Western Australia, Iran, Tanzania and Mauritius. The capacity of *P. margaritifera* to produce pearl colours overlapping the colour ranges of pearls of two other rival species is related to donor shell phenotypic diversity. This donor influence is observed not only at an inter-individual scale^13^ but also at an intra-individual level, through effects of the position on the donor mantle at which the graft is cut^14^.

**Figure 1:**
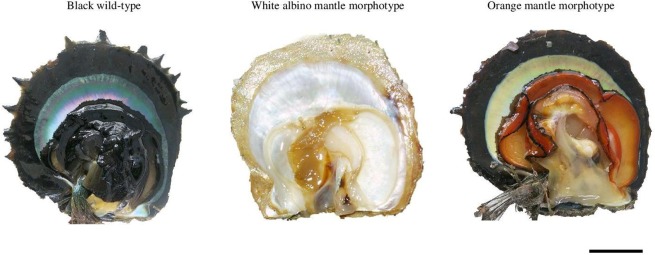
Coloured tissue and black morphotypes of *Pinctada margaritifera* originating from French Polynesia. From left to right: the black wild-type, white albino and orange-red morphotypes. Scale bars each represent 20 mm.

Molluscan shell colour is highly variable, and its richness has been studied in many species^15^. By contrast, only a few species display colour variation in their soft tissues. This is the case for some edible species which have high commercial value, as the colour of seafood flesh can be a key factor influencing consumer choice. The Pacific oyster (*Crassostrea gigas*) displays mantle edge colour variation, which was a candidate trait selected in the United States Molluscan Broodstock Program^16^. In this species, black, white and orange/ bronze and golden mantle colours exist^17^. In Korea, oysters with a black mantle are preferred and sell for a price about 20% higher^18^. The mussel *Mytilus californianus* has gonadic tissue colour differences, usually associated with sex, the female being orange and the males pale cream^19^, although tissue colours can also be influenced by ecological and environmental factors such as stress or nutrition in the intertidal zone^20^. In *P. margaritifera*, three distinct phenotypic colour morphs exist for the soft tissues: 1) the deep black wild-type phenotype (black mantle), 2) the albino phenotype (white mantle), and 3) the orange-red phenotype (orange–red mantle)^21–23^ (see Fig. 1). To date, only three previous studies have been made on these soft tissue colour morphotypes in *P. margaritifera*. One previous study, using the suppressive and subtractive hybridization (SSH) method, identified a set of genes differentially expressed between black phenotypes and full albino individuals^24^. The results revealed that nacre colour was partially under the influence of genes involved in three main genetic processes: 1) the biomineralization of the calcitic layer, 2) the formation of the aragonite tablets of the nacre layer, and 3) the biosynthesis chain of pigments such as melanin. Based on controlled crosses, flesh colour variation in *P. margaritifera* was found to be under genetic control and not related to environmental influence. The Mendelian inheritance of the orange morphotype was thus confirmed, with a two-allele model for the tissue colour trait in which the orange allele is recessive to the black one^25^. Study of genomic regions associated with the tissue colour difference between black and orange *P. margaritifera* morphotypes from Fijian sites identified five loci from tyrosinase pathways^26^. These pathways are known to play an important role in the formation of the shell matrix and melanin biosynthesis in mollusc tissues and shells^27,28^.

No previous studies had been made of the influence of the orange mantle morphotype on biomineralisation activities either in the shell or in cultured pearls produced using individuals of this morphotype as graft donors. To examine the phenotypic association, we made experimental grafts (N = 2000) using both orange mutant and black wild-type *P. margaritifera* as graft donors. Quantification of the darkness level of the pigmented inner shell of the donors, together with the qualitative phenotypic traits of the associated cultured pearl colour (darkness level, colour categories and lustre) were scored. A panel of candidate genes selected according to a previous study on mantle from white (albino) *P. margaritifera*^24^ was tested by RT-qPCR for their potential expression in both graft and pearl sac tissues from the orange mantle morphotype. These genes were selected for their: 1) putative implication in pigmentation (*tyr2a* and *zinc*), 2) implication in biomineralisation processes (*krmp7* and *shem4*), or 3) indirect role in the biomineralisation processes (*pdz* and *serp*). This preliminary molecular investigation aimed to examine whether the same pattern of gene expression implicated in the pathway determining white mantle could be associated with the orange mantle morphotype.

## Results

### Darkness of donor inner shell colour

**Figure 2:**
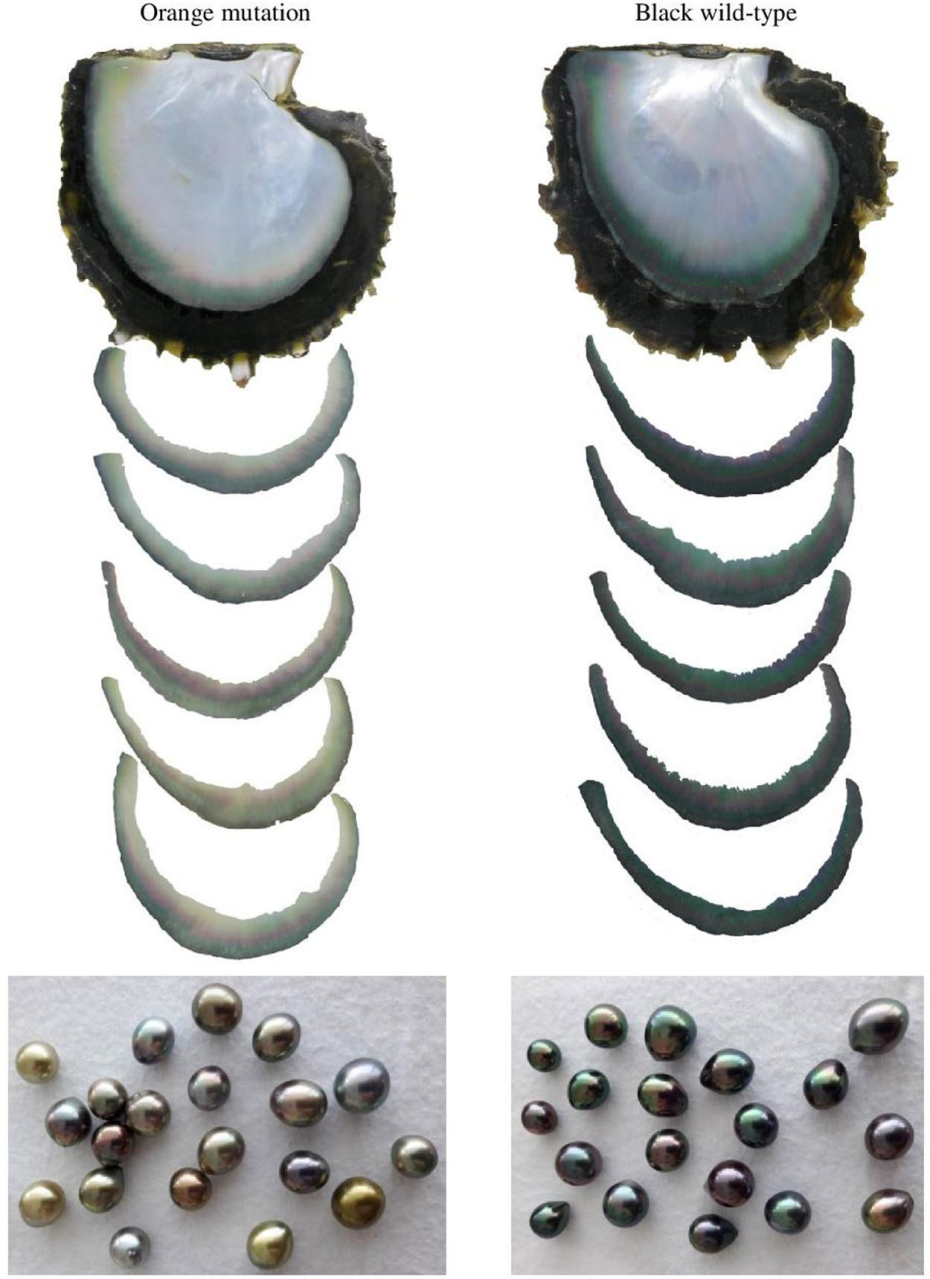
Inner shells of the two morphotypes of *P. margaritifera* (above) with associated grade A cultured pearls (below) produced using these morphotypes as donor oysters.

**Figure 3:**
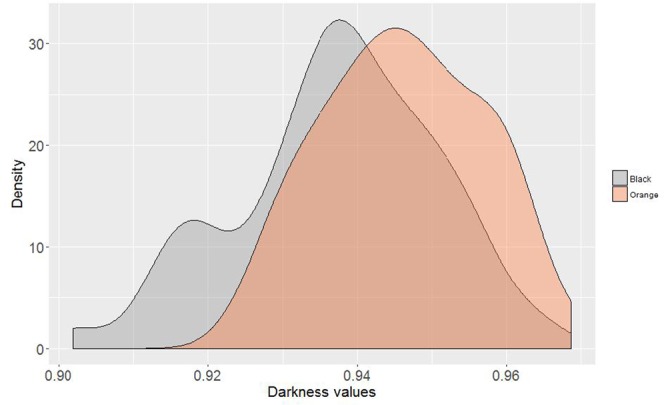
Density plot distribution of donor oyster inner shell darkness level in *P. margaritifera* for black wild-type and orange flesh phenotypes.

The inner shell colour band in *P. margaritifera* had paler visually-perceived colour in the orange mantle mutant, than in the black wild-type (Fig. 2). Quantification of the overall inner shell darkness level was analysed using the R package ImaginR. The corresponding distribution is shown in Fig. 3, with the darkness scale from 1 (lightest) to 0 (darkest). The average values of darkness level for the inner shell of the black wild-type and orange mutant were 0.937 and 0.946, respectively. The darkness distribution of the two donor phenotypes was significantly different (*p* = 0.0002; Wilcoxon followed by Hollander and Wolfe tests), with the inner shells of orange flesh mutant paler than those of the black wild-type.

### Experimental graft

When *P. margaritifera* with orange flesh was used as the donor, nucleus retention rate was 81.0% (N = 810). The remaining 18.6% corresponded to oysters that had rejected their nuclei (N = 186), and 0.4% mortality (N = 4). For the black wild-type flesh donors, the retention rate was 79.0% (N = 790), with another 20.0% of the oysters that had rejected their nuclei (N = 200) and 1.0% mortality (N = 10). No significant difference was found for retention rate between the two flesh colours. After 16 months of culture, 621 and 676 cultured pearls (non graded) were harvested from the black and orange donors, respectively. Differences observed between nucleus retention checking and harvest corresponded to mortalities, which were mainly due to predation. The main predators of *P. margaritifera* in French Polynesian lagoons are Triggerfish (*Balistoides viridescens*, *Pseudobalistes flavimarginatus*), diodonfish (*Diodon liturosus*), sea snails of the *Cymatium* genus, leopard-ray (*Aetobatus narinari*), and green sea turtle (*Chelonia mydas*).

### Variation in cultured pearl colour traits

Concerning the overall pearl darkness level, rates of pale-coloured pearls were four times more frequent (*p* < 0.001; χ² followed by Fisher’s exact test) when orange mantle donors were used than when black wild-type donors were used (Table 1). This was confirmed through assessment by professional GIE grading, where pale pearls were found at a higher proportion (×3) following grafting with orange mantle donors. A statistically significant difference was also noted for the medium darkness level category, as orange mantle donors produced twice as many in this group (Table 1). Inversely, donors with black mantle produced four times more dark pearls.

**Table 1:** Comparison between cultured pearl colours originating from *P. margaritifera* donors with black or orange flesh.

Cultured pearl traits		Black (N = 621)		Orange (N = 676)		Significance
Darkness	Light	8.53	(53)	32.1	(217)	***
	Medium	30.43	(189)	53.85	(364)	***
	Dark	61.03	(379)	14.05	(95)	***
Colour	Grey	33.33	(207)	60.65	(410)	***
	Green	26.41	(164)	23.37	(158)	NS
	Peacock	26.25	(163)	5.62	(38)	***
	Aubergine	1.45	(9)	1.92	(13)	NS
	Blue	5.15	(32)	0.15	(1)	***
	Yellow	6.92	(43)	7.1	(48)	NS
	White	0.48	(3)	1.18	(8)	NS
Colour (GIE)	Dark	44.64	(250)	46.3	(313)	NS
	Green	10.71	(60)	1.04	(7)	***
	Light	16.43	(92)	46.89	(317)	***
	Light Dark	28.21	(158)	5.77	(39)	***
Lustre	0	1.45	(9)	22.78	(154)	***
	1	26.09	(162)	70.41	(476)	***
	2	72.46	(450)	6.8	(46)	***

Nomenclature of the colour categories is described in the Materials and Methods section. Numbers indicate the proportion (%) and numbers in brackets correspond to frequencies. Traits significantly different between donor morphotype at *p* < 0.001 are indicated with 3 asterisks (*). NS: non-significant.

**Figure 4:**
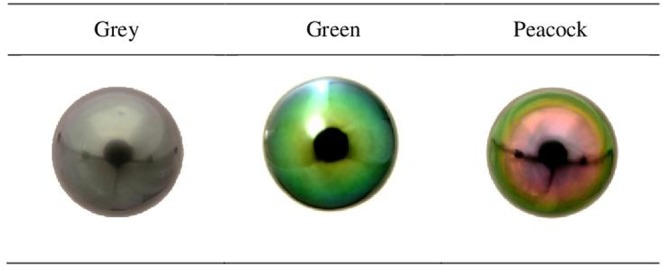
Cultured pearl colour categories produced by *Pinctada margaritifera* (grey, green and peacock). Picture samples concern round-shaped pearls.

The pearl colour categories grey, green and peacock (Fig. 4) were predominant and constituted from 86% to 90% of the total harvest (Table 1). The black mantle donors produced around five times more peacock pearls than did orange mutant donors. In contrast, the latter produced twice the number of grey pearls than the black wild-type donors. The GIE professional grading showed that the highest proportion of green pearls was found among pearls from black mantle donors.

Regarding pearl lustre, black wild-type donors produced a significantly higher proportion of pearls with high and medium degrees of lustre (99%) than donors with the orange mantle mutation (77%) (Table 1).

Figure 2 shows samples of harvested cultured pearls from the orange and black mantle phenotypes illustrating the darkness levels.

### Candidate gene expression

**Figure 5:**
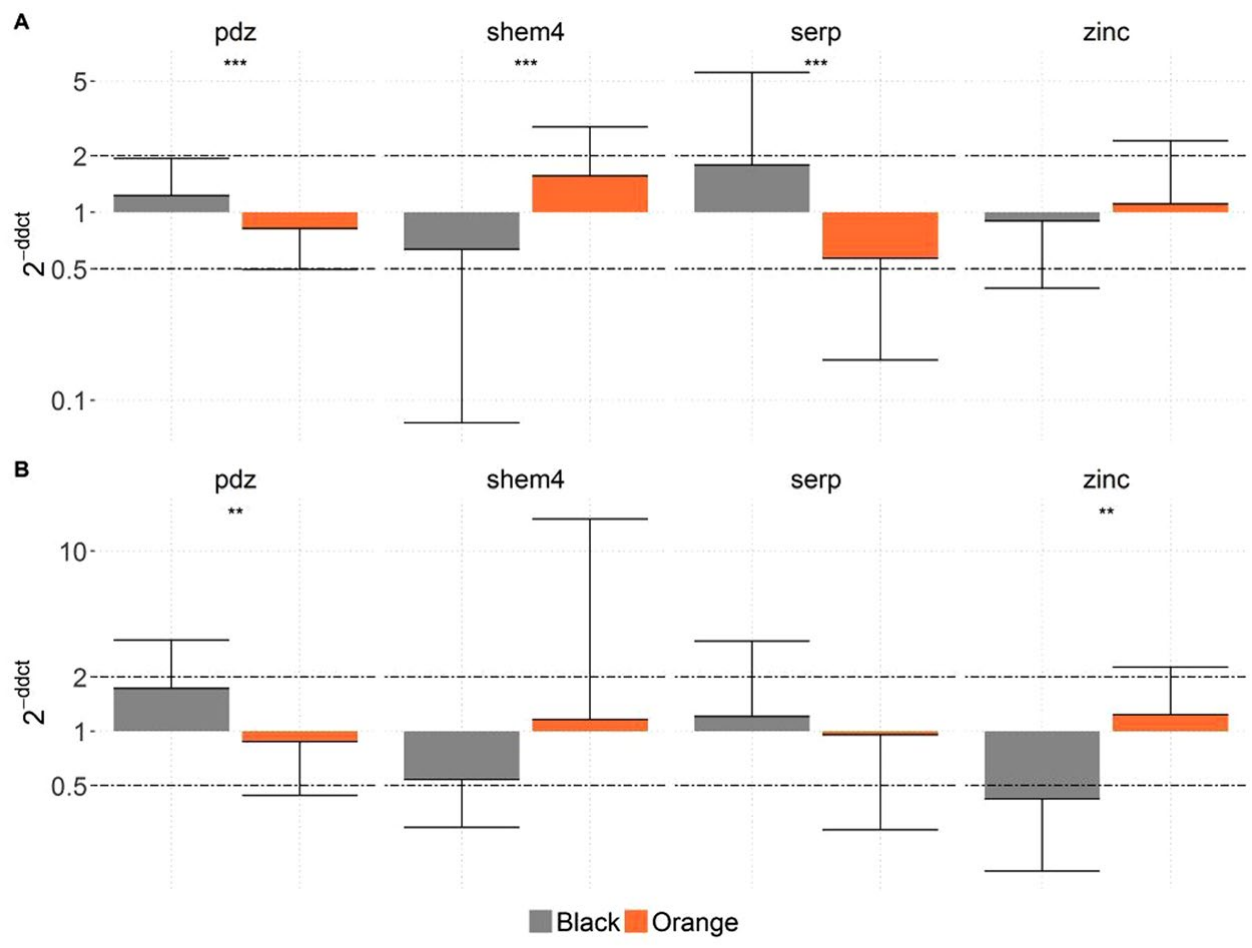
Relative expression of four biomineralization genes in *P. margaritifera* graft tissue (**A**) and pearl sac tissue (**B**). Grey bars represent data for the black-fleshed phenotype and orange bars represent data for the orange-fleshed phenotype. Y axes are in the logarithmic scale. Error bars indicate standard deviations. Statistical differences between the phenotypes are indicated by asterisks: ^*^for 0.05 > *p* > 0.005, ^**^for 0.005 > *p* > 0.0005, ^***^for *p* < 0.0005.

Significant relative expression for the panel of protein-coding genes implicated in calcite and aragonite layers in the graft and pearl sac tissue of the black and orange phenotypes was observed (Fig. 5). Out of the six candidate genes tested, only four were differentially expressed.

In graft tissue (Fig. 5A), expression of the genes *pdz*, *shem4* and *serp* was significantly different (*p* < 0.0005) between the two flesh colour phenotypes. *Shem4* had a higher relative gene expression ratio in orange mantle donors (2.45), while *pdz* and *serp* had higher relative gene expression ratios in the black wild-type (1.50 and 3.14 respectively).

In pearl sac tissue (Fig. 5B), the expression of *pdz* and *zinc* genes was significantly different (p < 0.005), with an over-expression of *zinc* in the orange mantle donor (2.93) and *pdz* in the black mantle donor (1.98).

Only *pdz* showed the same expression pattern in both tissues, with over-expression in the black wild-type *P. margaritifera* compared with the orange mutant (1.50 and 1.98 for graft tissue and pearl sac, respectively).

## Discussion

The relation between donor shell colour band and corresponding cultured pearl colour is well known in the *Pinctada* genus. In *P. fucata*, Wada and Komaru^29^ observed an association between donor shell and pearl colour. In reciprocal xenografts involving two *Pinctada* species that normally produce pearls with distinctively different base colours (*P. maxima*, the silver-lip pearl oyster, and *P. margaritifera*, the black-lip pearl oyster), McGinty *et al*.^30^ showed that the donor oyster is the primary determinant of pearl colour. Indeed, when a *P. maxima* donor was used, the resulting pearl had a white to silver base colour consistent with that of *P. maxima*, while *P. margaritifera* donors produced pearls with a grey to black base colour consistent with that of *P. margaritifera*, regardless of the recipient oyster species. By contrast, the link between donor mantle colour and pearls had yet to be examined. In the present study, colour quantification and qualification of the biomineralized structures (shell and cultured pearls, respectively) derived from *P. margaritifera* with the orange mantle morphotype revealed an associated paler colouration of both the inner shell colour band and cultured pearls (compared with the black wild-type). It is established that the implanted mantle graft from the donor and its derived pearl sac tissue play a significant role in the biomineralization process implicated in cultured pearl colour determination^8,30–32^. In *P. margaritifera*, the broad pearl colour range is the direct reflection of donor oyster shell colour diversity, particularly the chromatic inner shell band^17^. Heritability estimates for colour transmission have been recently published, signifying important donor oyster effects with a genetic basis^33^. Donor effect on pearl colour had already been described at the scale of individual wild donors and hatchery-bred families^12,34^. A statistical model could then be built to predict pearl colour categories at harvest following donor colour phenotype selection based on both inner and outer shell colour^35^. This was done by building a classification tree model, according to shell phenotype and culture location, using black and red outer shell phenotypes, combined with green and yellow inner shell phenotypes^35^. Colour transmission was visible from the spat stage, once again showing the genetic basis. This same study confirmed the role played by the donors in colour transmission, as all recipients used had the same black wild-type mantle phenotype. This relation can also be seen at an earlier stage between spat shell colour and pearl colour categories, with green/blue pearls obtained twice as often when using grey or green spat phenotypes as donors, and aubergine/peacock pearls obtained four times more often when using red or yellow spat phenotypes^36^. Some studies have reported a positive correlation between pigmentation of shell and mantle edge in other bivalve models, such as the Pacific oyster, *Crassostrea gigas*. Indeed, Kang *et al*.^18^ suggested that black and white mantle edge pigmentations in this species are heritable and correlated.

Four of the six candidate genes, selected based on a previous study on white mantle phenotype grafts^24^, were differentially expressed between the black wild-type and orange mantle morphotypes: *pdz*, *shem4*, *serp* in the graft tissue and *pdz* and *zinc* in the pearl sac. Comparisons of orange *vs*. black and white vs black mantle^24^ graft tissue, showed dissimilar expression profiles, suggesting that the molecular mechanisms for genes underlying biomineralization processes are not the same in the orange mantle morphotype as in the white mantle morphotype. The *zinc* gene, which is known to be indirectly involved in shell pigmentation (but not directly linked to the calcification process), was more highly expressed in the pearl sac of the orange phenotype compared with the black phenotype, and not in the graft. The *zinc* gene codes for the Zinc metalloprotease homolog of Tyrosinase related protein 1, which is known to be involved in the melanin biosynthesis pathway: a tyrosinase-like protein was detected in the periostracum layer of *P. fucata*^37^. The total or partial absence of biosynthesis of non- or partly functional melanin protein resulted, in the most extreme case, in the white albino phenotype^38,39^. Lemer *et al*.^24^ suggested that the albino mantle phenotype might overcompensate for a non-functional melanin protein by over-expressing the gene coding for this protein. To a lesser extent, This might also be the case for the orange phenotype, resulting in pale-coloured cultured pearls with a lower amount of black melanin pigment. Jabbour-Zahab *et al*.^40^, working on the mantle histochemistry of *P. margaritifera*, found a melanin-like pigment responsible for the black colouration of the shell and mantle tissue that may account for the colouration of individuals expressing the black morphotype. Surprisingly, *tyr2A*, a gene implicated in tyrosinase pathways, which is known to play an important role in the formation of the shell matrix and melanin biosynthesis in mollusc tissues and shells^27,28^, was not differentially expressed between the two morphotypes in either tissue examined. Shematrin proteins are a family of mollusc proteins exclusively expressed in the mantle, particularly in the edge region^41^. This was confirmed in the present study, where no significant expression of *shem4* was observed in the pearl sac tissue. Shematrin proteins are synthesized in the mantle edge and secreted into the prismatic layer of the shell, where they are thought to provide a framework for calcification and to be responsible for shell toughness^42^. In this study, the gene *shem4* showed high expression patterns in the graft tissue of the orange phenotype samples. This was the opposite of what had been found with the white albino mantle^24^, where *shem4* was down-expressed. These differences imply that *shem4* may play a different role in the formation of the calcitic layer and could explain the opposite expression pattern observed between black and orange phenotypes. Two other genes not directly involved in biomineralisation processes were differentially expressed in the present study. The gene *pdz* showed significantly higher expression levels in black phenotypes than in orange ones in both tissues. This was consistent with results from the previous study comparing black and albino phenotypes^24^. The gene *serp* coding for serine protease inhibitor or serpins showed low expression levels in the orange mantle phenotype in the graft tissue compared with the black wild-type. This result is the opposite of what was found in the albino type, which again suggests that the pathways are dissimilar.

From an aquaculture point of view, the possibility of increasing the proportion of paler-coloured pearls by using the orange mantle morphotype as donor, will enrich the colour range, which could in turn enlarge the market demand. Most Polynesian production aims to produce dark colour, to suit the Asian market demand. Indeed, from the last economic data provided from cultured pearls produced in French Polynesia, exportation was mainly to China (62% of the total production) and Japan (32%)^43^. There is potential to develop American and European markets by increasing the availability of lighter coloured pearls. While pearl oysters with orange mantle are only found at low frequencies in French Polynesia, selection and mass production of such phenotypes in hatcheries could be used to pursue this potential market t and fulfil its potential demands. Such hatchery management to establish an orange mantle line must guarantee the genetic diversity and effective population sizes, as well as to reduce the level of inbreeding throughout production. In many hatchery systems for mass spawning species, the controlled rearing of the species through its entire life cycle, maintenance of pedigree, equalization of parental sex ratios and use of genetic markers to monitor genetic diversity over successive generations have become generalised.

This study, together with previous results on the white mutant^24^, are the first reported investigations of mantle morphotype associations in *P. margaritifera*. Such information on the mechanisms underlying mantle tissue colour is valuable for future mass assisted selection (MAS) and identification of quantitative trait loci (QTL) in this species. The orange mantle mutation of *P. margaritifera* is responsible for paler pigmentation levels observed in shells and in cultured pearls when individuals with this mutation are used as donors. Some gene expression patterns already known to be associated with the extreme white mantle mutation, which produces both shells and pearls with no pigmentation, were found to be associated with pale shell and pearl phenotypes in the orange morphotype examined here. These initial candidate gene expression studies open the way for the application of next generation sequencing technologies such as RNA-Seq, which would provide more information about the transcriptome associated with the biological mechanisms linked to the orange mantle morphotype, as was recently done to improve understanding of the molecular mechanisms determining cultured pearl quality traits^44^.

## Materials and Methods

### Specimen collection, grafting procedure and tissue sampling

Wild donor *Pinctada margaritifera* between 8–9 cm in dorso-ventral length were selected for their flesh colour: the black wild-type (N = 1000) and the rare orange morphotype (N = 200) (see Fig. 1). These individuals were collected as spat using commercial collectors in the Mangareva lagoon (Gambier Archipelago, French Polynesia), where the orange morphotype is more frequent than in other locations^25^. The animals were transferred by plane to the Ifremer facilities at Vairao on Tahiti Island (Society Archipelago, French Polynesia) in April 2014. They were left to acclimate for 2 months and then transferred to Arutua atoll (Tuamotu Archipelago, French Polynesia), where the experimental grafts would be performed. Oysters were deployed on long lines and maintained at a depth of around 6 m for another two months prior to grafting.

All grafts were performed by a single expert in Arutua atoll, in such a way as to minimize grafter effect on pearl quality traits. Inner shell colour phenotype of each donor oyster was checked using a speculum to open the valves. A dentist’s mirror was then inserted into the open oyster to view the inner shell colouration, particularly in the contact area with the mantle at the edge of the shell (band colour). For each flesh colour phenotype, 50 individuals with strongly coloured bands were selected. These 100 donors were then used to perform 2000 grafts (20 grafts per donor) using 2.2 BU nuclei (5.45 mm diameter; Imai Seikaku Co. Ltd., Japan). The recipient oysters (between 8–9 cm in dorso-ventral measurement) were obtained from local wild spat collection. Recipient oysters were chosen to be strong and healthy, which can be judged by the colour of the visceral mass and gills (brilliant appearance), shell size and appearance (round shape suggesting regular growth), and muscle resistance shown when opening the shells for the graft. Grafted oysters were then put into individual mesh retention bags and identified with labels according to the donor phenotype and individual donor oyster, allowing cases of nucleus rejection to be identified. Recipient oysters were examined 45 days post grafting to estimate nucleus retention rates and mortalities as previously described^45^. Donor oysters that had retained their grafted nucleus were grown on long lines and washed every four months with a high pressure spray (to remove epibiota).

In order to assess expression levels of known biomineralisation genes in the donor tissue and pearl sacs subsequently formed in the recipients, 3 to 5 pieces of graft tissue from each donor oyster were sampled during the grafting process, and one pearl sac per original donor was sampled at harvest. In order to minimize the inclusion of recipient tissues with those of the sampled pearl sacs, the latter were excised from recipient oysters by removing the outer layers with a surgical blade until only a thin (<0.5 mm) layer of tissue surrounding the pearl remained. This was immediately transferred into 2.0-ml tubes and preserved. in RNAlater® and stored at −80 °C until subsequent RNA extraction.

### Inner shell and cultured pearl colour measurements

The shells of the donor oysters were cleaned and stored away from light. The donors and pearl boxes were photographed with a Canon® PowerShot G9 at maximum pixel resolution into a Packshot Creator™ (v. 3.0.3.8) to prevent dark shadows and light reflection. The pictures of the donor’s inner shells were cut to extract the chromatic zone and the visible side of the pearl was also cut out (only one side of the pearl was photographed, we treat this as a colour sample of the pearl) using the free GNU Image Manipulation Program (version 2.8.22) software (selection with lasso, copy, paste like image, export in.jpeg format). ImaginR (V.2) package on R (3.1.3) was used to describe the overall darkness level of the inner shell side of the donors.

The harvested cultured pearls were cleaned by ultrasonication in soapy water (hand washing) with a LEO 801 laboratory cleaner (2-L capacity, 80 W, 46 kHz) and then rinsed in distilled water. Some *keshi* (small non-nucleated nacre deposits) were also harvested, but these were not graded in the present study. Colour evaluation was made by the naked eye without a jeweller’s loupe. Cultured pearls were classified according to their: 1) darkness of overall coloration, with three categories depending on the level: dark, medium and light; 2) visually-perceived colour category (due to pigments, which produce the bodycolor, and secondary colour, known as overtone), by two types of grader (see below); and 3) lustre, with 3 categories, no lustre (0), medium (1) and high (2). For colour categories, two sets of data were collected: 1) a professional grader from GIE Poe O Rikitea, according to the Tahitian pearl auction classification categories^43^, and 2) classic evaluation as previously described^46^.

### Gene expression analysis

The relative gene expression profiles from graft and pearl sac tissues were analysed for a set of candidate genes selected based on a previous study^24^. These genes were selected for their: 1) putative implication in pigmentation (*tyr2a* and *zinc*), 2) implication in biomineralisation processes (*krmp7* and *shem4*), and 3) indirect role in the biomineralisation processes (*pdz* and *serp*). Primer sequences of the six biomineralisation genes can be found in Supplementary Table S1. Some gene ontology information is given in Supplementary Table S2. Two genes were used as housekeeping genes, chosen based on their ubiquitous and constitutive expression patterns in *P. margaritifera* tissue: SAGE (SAGES: AGCCTAGTGTGGGGGTTGG/SAGER: ACAGCGATGTACCCATTTCC) (called REF)^47^ and GAPDH^24^ (GAPDHS: AGGCTTGATGACCACTGTCC/ GAPDHR: AGCCATTCCCGTCAACTTC). The relative stability of the GAPDH and SAGE combination was confirmed using NormFinder (stability value for best combination).

After removing the RNAlater® by pipetting and absorption, total cellular RNA was extracted from either the individual graft tissue or pearl sac sample using TRIzol® reagent (Life Technologies), according to the manufacturer’s recommendations. RNA was quantified using a NanoDrop ND-1000 spectrophotometer (NanoDrop Technologies, Inc.) and the quality of the RNA was checked to exclude degradation using an Agilent 2100 bioanalyser. The RIN values were between 6.50 and 7.40, indicating sufficient quality for quantitative real-time PCR analysis. Total RNA for each individual was then treated with DNAse I using a DNA-free Kit (Ambion). First strand cDNA was synthesized from 500 ng total RNA using the Transcriptor First Strand cDNA Synthesis Kit (Roche) and a mix of poly (dT) and random hexamer primers. Real-Time PCR amplifications were carried out on a Roche Light Cycler® 480. A no-RT control was screened by qPCR using a housekeeping gene to ensure there was no DNA contamination. The amplification reaction contained 5 μL LC 480 SYBR Green I Master Mix (Roche), 4 μL cDNA template, and 1 μL of primer (1 µM), in a final volume of 10 μL. Each run included a positive cDNA control and a blank for each primer pair. The run protocol was as follows: initial denaturation at 95 °C for 10 min followed by 40 cycles of denaturation at 95 °C for 30 s, annealing at 60 °C for 30 s and extension at 72 °C for 60 s. Lastly, the amplicon melting temperature curve was analysed using a melting curve program: 45–95 °C with a heating rate of 0.1 °C s^−1^ and continuous fluorescence measurement. All measurements were made in duplicate and all analyses were based on the Ct values of the PCR products. We permitted a difference of less than 0.5 Ct between our two replicates. If the difference was superior to 0.5 Ct, the qPCR reaction was repeated and, if congruent ct values were still not obtained, then the sample was removed. The relative expression ratio (R) of a target gene was calculated based on E and the CP deviation of an unknown sample versus a “control” and expressed in comparison to a reference gene as follows^48^: R = E_(target)_^ΔCt target (control − sample)^/E_(ref)_^ΔCt ref (control − sample)^. Here, the control represents the mean of the values obtained for the tested gene^49^. PCR efficiency (E) was estimated for each primer pair by determining the slopes of standard curves obtained from a serial dilution analysis of cDNA to ensure that E ranged from 90 to 110%.

### Statistical analysis

All analyses were performed using R© version 3.4.2 software (R foundation for Statistical Computing). The significance threshold was set at p ≤ 0.05. For qualitative categories such as colour, darkness, lustre, and GIE colour differences, χ² tests and Fisher’s exact test were used to detect differences between results obtained with black and orange donors, when an expected value < 5 was found.

The drift between the darkness distribution of black and orange donor inner shell colouration was test by using a Wilcoxon test (*stats v3.5.0* R package based on Hollander and Wolfe^50^ and Patrick Royston^51^) and a confidence interval based on Bauer^52^.

Gene expression was analysed using non parametric Kruskall-Wallis tests to test the effects of different coloured donor oysters.

## Supplementary information

Table S1 and Table S2

## Data Availability

The authors declare that all data are available.

## References

[1] Yukihira H, Lucas JS, Klumpp DW (2000). Comparative effects of temperature on suspension feeding and energy budgets of the pearl oysters *Pinctada margaritifera* and *P. maxima*. Marine Ecology Progress Series.

[2] L. Cunha R, Blanc F, Bonhomme F, Arnaud-Haond S (2010). Evolutionnary patterns in pearl oysters of the Genus *Pinctada* (*Bivalvia*: *Pteriidae*). Marine Biotechnology.

[3] Ky CL, Quillien V, Broustal F, Soyez C, Devaux D (2018). Phenome of pearl quality traits in the mollusc transplant model *Pinctada margaritifera*. Scientific Reports.

[4] Gervis, M. H. & Sims, N. The biology and culture of pearl oysters (*Bivalvia*: *Pteriidae*). *Overseas Development Administration and International Center for Living Aquatic Resources Management* 3–38 (1992).

[5] Southgate, P. C. *et al*. The Pearl Oyster (ed. Southgate, P. C. & Lucas, J. S.) (Elsevier 2008).

[6] Landman, N. H., Mikkelsen, P. M., Bieler, R. & Bronson, B. Pearls, a natural history. American Museum of Natural History (ed. Harry, N.) (Abrams Inc., New York 2001).

[7] Kishore, P. & Southgate, P. C. A detailed description of pearl-sac development in the black-lip pearl oyster, *Pinctada margaritifera* (*Linnaeus* 1758). *Aquac. Res*, 10.1111/are.12674 (2014).

[8] Blay, C., Planes, S. & Ky, C.-L. Donor and recipient contribution to phenotypic traits and the expression of biomineralisation genes in the pearl oyster model *Pinctada margaritifera*. *Scientific Reports*. **7**(1), 2696 (1–12) (2017). 10.1038/s41598-017-02457-x PMC5457395 28578397

[9] Taylor, J. Producing golden and silver south sea pearls from indonesian hatchery reared *Pinctada maxima*. *SPC Pearl Oyster Information Bulletin*, 30 (2002).

[10] Tong Y, Shen H (2001). Quality assessment and testing of akoya pearls. China Gems and Jades.

[11] Karampelas S, Fritsch E, Gauthier JP, Hainschwang T (2011). Uv-vis-nir reflectance spectroscopy of natural-colour saltwater cultured pearls from *Pinctada margaritifera*. Gems and Gemology.

[12] Ky CL (2013). Family effect on cultured pearl quality in black-lipped pearl oyster *Pinctada margaritifera* and insights for genetic improvement. Aquatic Living Resource.

[13] Ky CL, Lo C, Planes S (2017). Mono- and polychromatic inner shell phenotype diversity in *Pinctada margaritifera* donor pearl oysters and its relation with cultured pearl colour. Aquaculture.

[14] Ky CL (2018). Variation in cultured pearl quality traits in relation to position of saibo cutting on the mantle of black-lipped pearl oyster *Pinctada margaritifera*. Aquaculture.

[15] Williams, S. T. Molluscan shell colour. *Biological Reviews* (2016). 10.1111/brv.12268 27005683

[16] Brake J, Evans F, Langdon C (2004). Evidence for genetic control of pigmentation of shell and mantle edge in selected families of Pacific oysters. Crassostrea gigas. Aquaculture.

[17] Nell JA (2001). The history of oyster farming in Australia. Mar. Fish. Rev..

[18] Kang J-H (2013). Characterizations of Shell and Mantle Edge Pigmentation of a Pacific Oyster, *Crassostrea gigas*, in Korean Peninsula. Asian-Australasian Journal of Animal Sciences.

[19] Joelley Jolley DF, Maher WA, Kyd J (2004). Selenium accumulation in the cockle *Anadara trapezia*. Environ. Pollut..

[20] Petes LE, Menge BA, Chan F, Webb MAH (2008). Gonadal tissue color is not a reliable indicator of sex in rocky intertidal mussels. Aquatic Biology.

[21] Shirai, S. & Nakamura, S. Pearls and Pearl Oysters of the World. Marine Planning Co., Okinawa, Japan (1994).

[22] Hwang JJ, Okutani T (2003). Taxonomy and distribution of the genera Pteria and Pinctada (Bivalvia: Pteriidae) in Taiwan. J. Fish. Soc. Taiwan.

[23] Wada, K. T. & Tëmkin, I. The Pearl Oyster, Taxonomy and Phylogeny: Commercial Species (ed. P.C. Southgate, P.C. & Lucas J.S.) (Elsevier 2008).

[24] Lemer S, Saulnier D, Gueguen Y, Planes S (2015). Identification of genes associated with shell color in the black lipped pearl oyster, *Pinctada margaritifera*. BMC Genomics.

[25] Ky CL, Nakasai S, Pommier S, Sham Koua M, Devaux D (2016). The Mendelian inheritance of rare flesh and shell colour variants in the black-lipped pearl oyster (*Pinctada margaritifera*). Animal Genetics.

[26] Lal MM, Southgate PC, Jerry DR, Zenger KR (2016). Fishing for divergence in a sea of connectivity: The utility of ddRADseq genotyping in a marine invertebrate, the black-lip pearl oyster *Pinctada margaritifera*. Marine Genomics.

[27] Nagai K, Yano M, Morimoto K, Miyamoto H (2007). Tyrosinase localization in mollusc shells *Comp. Biochem*. Physiol. B: Biochem. Mol. Biol..

[28] Takgi, R. & Miyashita, T. A cDNA cloning of a novel alpha-class tyrosinase of *Pinctada fucata*: its expression analysis and characterization of the expressed protein. *Enzym. Res*. 9 (2014). 10.1155/2014/780549 PMC4003781 24818013

[29] Wada KT, Komaru A (1996). Color and weight of pearls produced by grafting the mantle tissue from a selected population for white shell color of the japanese pearl oyster *Pinctada fucata martensii* (Dunker). Aquaculture.

[30] McGinty EL, Evans BS, Taylor JUU, Jerry DR (2010). Xenografts and pearl production in two pearl oyster species, *P. maxima* and *P. margaritifera*: effect on pearl quality and a key to understanding genetic contribution. Aquaculture.

[31] Arnaud-Haond S (2007). Pearl Formation: Persistence of the Graft During the Entire Process of Biomineralization. Marine Biotechnology.

[32] Zhifeng (2014). Contribution of donor and host oysters to the cultured pearl colour in *Pinctada martensii*. Aquaculture Research.

[33] Blay, C., Planes, S. & Ky, C.-L. Crossing phenotype heritability and candidate gene expression in grafted black-lipped pearl oyster *Pinctada margaritifera*, an animal chimera. *Journal of Heredity*, 10.1093/jhered/esy015 (2018). 10.1093/jhered/esy015 29584922

[34] Tayale A (2012). Evidence of donor effect on cultured pearl quality from a duplicated grafting experiment on *Pinctada margaritifera* using wild donors. Aquatic Living Resources.

[35] Ky C-L (2017). Is pearl colour produced from *Pinctada margaritifera* predictable through shell phenotypes and rearing environments selections?. Aquaculture Research.

[36] Ky C-L, Sham Koua M, Le Moullac G (2018). Impact of spat shell colour selection in hatchery-produced *Pinctada margaritifera* on cultured pearl colour. Aquaculture Reports.

[37] Zhang C, Xie L, Huang J, Chen L, Zhang R (2006). A novel putative tyrosinase involved in periostracum formation from the pearl oyster (*Pinctada fucata*). Biochem Biophys Res Commun..

[38] King RA, Summers CG (1998). Albinism. Dermatol Clin..

[39] Oetting WS, King RA (1999). Molecular basis of albinism: mutations and polymorphisms of pigmentation genes associated with albinism. Hum Mutat..

[40] Jabbour-Zahab R, Chagot D, Blanc F, Grizel H (1992). Mantle histology, histochemistry and ultrastructure of the pearl oyster *Pinctada margaritifera* (L.). Aquat. Living Resour..

[41] Marie B (2012). Characterization of MRNP34, a novel methionine-rich nacre protein from the pearl oysters. Amino Acids.

[42] Yano M, Nagai K, Morimoto K, Miyamoto H (2006). Shematrin: a family of glycine-rich structural proteins in the shell of the pearl oyster *Pinctada fucata*. Comp Biochem Physiol B Biochem Mol Biol..

[43] Ky, C.-L., Broustal, F., Potin, D. & Lo, C. The pearl oyster (*Pinctada margaritifera*) aquaculture in French Polynesia and the indirect impact of long-distance transfers and collection-culture site combinations on pearl quality traits. *Aquaculture Reports*, **13**10.1016/j.aqrep.2019.100182 (2019).

[44] Le Luyer, J. *et al*. Whole transcriptome sequencing and biomineralization gene architecture associated with cultured pearl quality traits in the pearl oyster, *Pinctada margaritifera*. *BMC genomics*, in press (2019). 10.1186/s12864-019-5443-5 PMC6366105 30727965

[45] Ky C-L, Molinari N, Moe E, Pommier S (2014). Impact of season and grafter skill on nucleus retention and pearl oyster mortality rate in *Pinctada margaritifera* aquaculture. Aquaculture International.

[46] Ky C-L, Okura R, Nakasai S, Devaux D (2016). Quality Trait Signature at Archipelago Scale of the Cultured Pearls Produced by the Black-Lipped Pearl Oyster (*Pinctada margaritifera* Var. *cumingi*) in French Polynesia. Journal Of Shellfish Research.

[47] Joubert, C. *et al*. Temperature and Food Influence Shell Growth and Mantle Gene Expression of Shell Matrix Proteins in the Pearl Oyster *Pinctada margaritifera*. Plos One http://journals.plos.org/plosone/article?id=10.1371/journal.pone.0103944 (2014). 10.1371/journal.pone.0103944 PMC4133174 25121605

[48] Pfaffl MW (2001). A new mathematical model for relative quantification in real-time RT–PCR. Nucleic acids research.

[49] Andersen CL, Jensen JL, Ørntoft TF (2004). Normalization of real-time quantitative reverse transcription-PCR data: a model-based variance estimation approach to identify genes suited for normalization, applied to bladder and colon cancer data sets. Cancer Research.

[50] Hollander, M., A Wolfe, D. & Chicken, E. The one‐way layout. Nonparametric Stat. Methods, Third Ed. 202–288 (1973).

[51] Royston, P. Remark AS R94: A remark on algorithm AS 181: The W-test for normality. J. R. Stat. Soc. Ser. C (Applied Stat. 44, 547–551 (1995).

[52] Bauer DF (1972). Constructing confidence sets using rank statistics. J. Am. Stat. Assoc..

