# Supplementary material for: Relationship of the orange tissue morphotype with shell and pearl colouration in the mollusc Pinctada margaritifera
Source: Sci Rep. 2019 Mar 26;9:5114. doi: 10.1038/s41598-019-41581-8 (PMC6435800; doi:10.1038/s41598-019-41581-8)
Supplement: Supplementary file 1 — Table S1 and Table S2 [file 41598_2019_41581_MOESM1_ESM.docx]

**Supplementary information**

**Title of the manuscript:**

**Relationship of the orange tissue morphotype with shell and pearl colouration in the mollusc *Pinctada margaritifera***

**Full author list:**

Chin-Long KY, Carole BLAY, Floriane BROUSTAL, Manaarii SHAM KOUA, Serge PLANES

**Supplementary table S1**. Set of forward and reverse primers used for gene expression analysis by quantitative RT-PCR in graft and pearl sac tissues of *Pinctada margaritifera*.

| Primer name | GenBank  Accession numbers | Forward primer (5'-3') | Reverse primers (3'-5') |
| --- | --- | --- | --- |
| pdz | JZ845584 | TGAGCTTCAGAGAGGTGACG | AACATTTGGTGAGGGTTTGG |
| shem4 | JZ845594 | GCTTCCCATCGGTTTATGG | TGCCAACATTTCCGTATCC |
| serp | JZ845608 | AGGTGTGTACCATTCTTCTACGG | GCAAACATCTCCTCCATCTCC |
| zinc | JZ845609 | CAGAGATGGTTTTGTGTTACTTACG | GCTTTGAGGCATTCATGTCC |
| krmp7 | JZ845792 | GCCTTCACCACAGAAGGAAG | GCCGAATTTCTTCAGACACC |
| tyr2A | JZ845579 | GCGGCTCTACTGTCAAATGG | CTGGACCTTTCAGGGACTGG |

**Supplementary table S2**. Candidate gene ontology informations through Uniprot database search. First line for each gene correspond to accession number, second line to name, third line to ontology and fourth line to synonyms.

| Gene | Ontology information | | |
| --- | --- | --- | --- |
| tyr2A | GO:0005576  extracellular region  cellular_component  extracellular | GO:0046872  metal ion binding  molecular_function  metal binding, heavy metal binding | GO:0016491  oxidoreductase activity  molecular_function  redox activity, oxidoreductase activity, acting on other substrates |
| serp | GO:0005576  extracellular region  cellular_component  extracellular | GO:0004867  serine-type endopeptidase inhibitor activity  molecular_function  serine protease inhibitor activity, serpin |  |
| zinc | GO:0005576  extracellular region  cellular_component  extracellular | GO:0004222  metalloendopeptidase activity  molecular_function  metalloendoprotease activity | GO:0008270  zinc ion binding  molecular_function  zinc binding |
